# A Wireless Swing Angle Measurement Scheme Using Attitude Heading Reference System Sensing Units Based on Microelectromechanical Devices

**DOI:** 10.3390/s141222595

**Published:** 2014-11-27

**Authors:** Bingtuan Gao, Zhenyu Zhu, Jianguo Zhao, Boran Huang

**Affiliations:** 1 School of Electrical Engineering, Southeast University, No. 2, Sipailou, Nanjing 210096, China; E-Mails: zzy787232500@163.com (Z.Z.); hbr112358@163.com (B.H.); 2 Department of Electrical and Computer Engineering, Michigan State University, East Lansing, MI 48824, USA; E-Mail: zhaojia1@msu.edu

**Keywords:** swing angle, crane, microelectromechanical system (MEMS), attitude heading reference system (AHRS), wireless

## Abstract

Feasible real-time swing angle measurement is significant to improve the efficiency and safety of industrial crane systems. This paper presents a wireless microelectromechanical system (MEMS)-based swing angle measurement system. The system consists of two attitude heading reference system (AHRS) sensing units with a wireless communication function, which are mounted on the hook (or payload) and the jib (or base) of the crane, respectively. With a combination of a three-axis accelerometer, a three-axis gyroscope and a three-axis magnetometer, the standard extended Kalman filter (EKF) is used to estimate the desired orientation of the payload and the base. Wireless ZigBee communication is employed to transmit the orientation of the payload to the sensing unit mounted on the base, which measures the orientation of the base. Because several physical parameters from the payload to the base can be acquired from the original crane control system, the swing angles of the payload can be calculated based on the two measured orientation parameters together with the known physical parameters. Experiments were performed to show the feasibility and effectiveness of the proposed swing angle measurement system.

## 1. Introduction

Industrial cranes are widely used in harbors, docks, warehouses, construction sites and other industrial sites. They are desired for transferring goods in a fast and safe way. However, due to the acceleration of the cart, the swinging of the payload is inevitable. Therefore, it is necessary to anti-sway control the crane with experienced operators or an automatic control system. To suppress the payload swing of crane systems, many schemes have been developed over recent years [1–4]. Generally, the developed schemes could be categorized into open-loop anti-swing control and closed-loop anti-swing control. The open-loop anti-swing control mainly includes optimal time control [5] and input shaping control [6]. The key disadvantage of the open-loop anti-swing control is that the control system is not robust with the parameter changing and unforeseen disturbances. The closed-loop anti-swing control scheme could achieve better trajectory control performance and robustness on parameter variation and unforeseen disturbances. However, closed-loop anti-swing control schemes are based on feedback information, especially the swing angles [2,3,7,8].

Currently, some researchers have estimated or observed the swing angles, other than measuring them with sensors directly. For example, Gholabi *et al.* [1] employed the voltage and current of the induction motor, which drives the trolley, to estimate swing angles for damping control of the payload swing. Ebrahimi *et al.* [9] estimated the swing angles based on the load cell installed in the trolley for feedback control of the overhead crane. To measure the swing angles directly, they can be acquired based on optical encoders [10], potentiometers [5], cameras [11–14], accelerometers or inclinometers [15,16] and other types of sensors. Generally, optical encoder- and potentiometer-based angle measurement could be suitable only for a lab crane setup. Vision-based swing angle measurement suffers from high cost and the industrial environment. Accelerometer or inclinometer sensors are competitive for swing angle measurement, especially with the development of the MEMS technology, which leads to low cost accelerometers or inclinometers. Once these accelerometers or inclinometers are attached to some special locations (normally a hook or trolley) of the crane body, the swing angles can be calculated based on the geometry of the crane system. The flaw of using an accelerometer or inclinometer only to measure the swing angles is that the payload will rotate along the rope in some cases. Therefore, the orientation sensors should meet all of the cases.

Orientation estimation systems have been widely applied in airplanes, virtual reality, game systems, robotic systems, unmanned aerial vehicles, *etc.* For example, an aerial vehicle needs orientation information to stabilize its attitude [17]. Traditionally, such sensing systems are very expensive, because they use high resolution and low bias sensors, and they usually find applications in commercial airplanes. With the advancement of MEMS technology, various low-cost motion sensors are widely available nowadays. The output of these sensors, however, has a low resolution, high noise and a time-varying bias term. Therefore, sophisticated fusion algorithms are needed to estimate the orientations. Some AHRS sensors are already available in the market nowadays. For example, the AHRS440 from Crossbow Technology [18], the 3DM-GX3-25 from Microstrain [19], the VN-100 from VectorNav [20], the MTi from Xsens [21], InertiaCube from Intersense [22], ChR-6dm from CHRobotics [23], *etc.* Most of them are quite expensive, except the VN-100 from VectorNav and ChR-6dm from CHRobotics. Moreover, ChR-6dm from CHRobotics is open source, which means we can modify the firmware on it.

In this paper, two MEMS-based AHRS sensors will be used to calculate the swing angles of the crane system. The two sensors are installed on the hook (or payload) and the jib (or crane body) to measure the orientation of the payload and the crane body, respectively Based on the measured parameters, swing angles of the payload can be calculated together with geometrical parameters of the crane system.

The rest of the paper is organized as follows. The swing angle measurement system will be described in Section 2. After that, in Section 3, the deduction of swing angles with two MEMS-based AHRS sensors will be presented in detail, including static orientation calculation, orientation update and swing angles calculation. The experimental setup and implementation of the swing angles measurement scheme will be demonstrated and discussed in Section 4. Finally, conclusions and future work will be summarized in Section 5.

## 2. The Proposed Swing Angles Measurement Scheme

**Figure 1.**
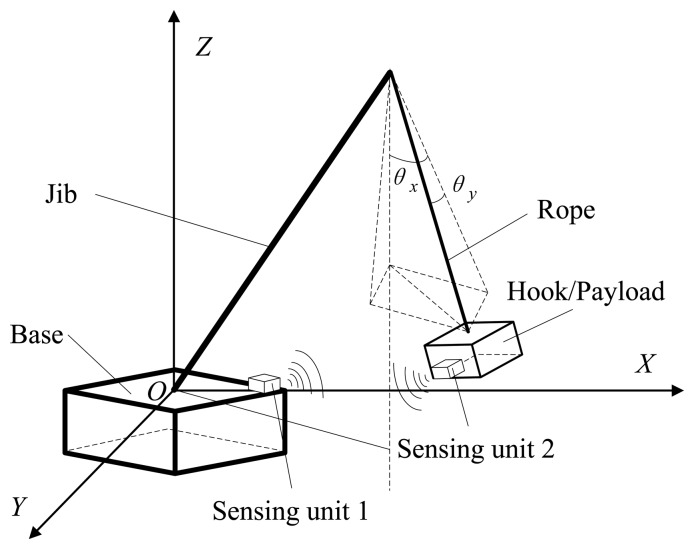
Swing angles measurement using wireless sensing units for crane systems.

The proposed swing angles measurement scheme for a mobile crane system can be described as shown in Figure 1. The swing motion of the payload of other types of crane systems can be described similarly. Inertial coordinates *OXYZ* are attached on the base, which means the crane body. The spatial swing angles of the payload could be denoted with two plane swing angles *θ_x_* and *θ_y_*. Our task is to acquire the real-time dynamic data of the two angles for monitoring and anti-swing control of the payload.

The angles measurement scheme is to use two MEMS-based AHRS sensors with a wireless communication function, as shown in Figure 1. Sensing Unit 1 can be installed near the control box of the crane system, and Sensing Unit 2 is installed on the hook or the payload. Each sensing unit can measure the orientation of the object on which it is fixed. We denote the orientation of the payload as “body” coordinates and the orientation of the crane base as “inertial” coordinates. Sensing Unit 2 will send its orientation, *i.e.*, pitch (*θ*), roll (*ϕ*) and yaw (*ψ*) angles, to Sensing Unit 1, which measures the orientation of the crane body. As the orientation of the top end of the jib can be calculated with the known parameters of the crane and the orientation of the base, the two swing angles *θ_x_* and *θ_y_* therefore can be calculate based on the two measured orientation angles of the base and the payload together with the physical parameters of the crane. The calculated swing angles *θ_x_* and *θ_y_* then could be sent to the control box of the crane system through cabled serial communication for monitoring and/or closed-loop anti-swing control design.

**Figure 2.**
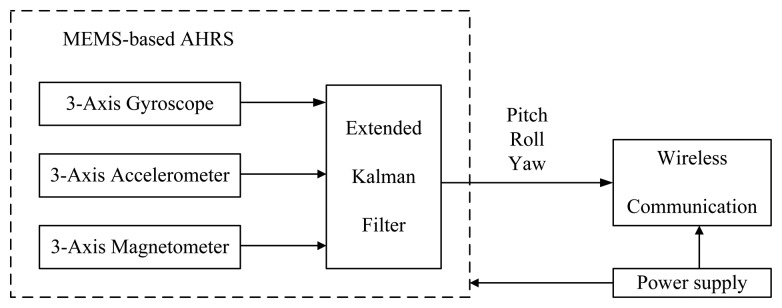
Diagram illustration of each wireless MEMS-based AHRS sensing unit.

The diagram of each sensing unit can be illustrated as shown in Figure 2. The AHRS sensor is mainly based on three MEMS devices: three-axis accelerometer having output a = [*a_x_*, *a_y_*, *a_z_*]^T^, three-axis magnetometer having output h = [*h_x_*, *h_y_*, *h_z_*]^T^ and three-axis gyroscope having output *ω* = [*ω_x_*, *ω_y_*, *ω_z_*]^T^. The sensing signals from the devices will be integrated to estimate the orientation (pitch, roll, yaw angles) based on fusion algorithms, such as EKF. Additionally, the pitch (*θ*), roll (*ϕ*) and yaw (*ψ*) angles measured by one sensing unit can be sent to other units through wireless communication in real time. The advantages of the designed sensing unit are the low cost, compact size, low energy consumption, robustness to the different weather environments, and so on.

## 3. Derivation of Swing Angles

The derivation of swing angle measurement for crane systems includes three key steps:

(1) initializing the orientation of each AHRS sensing unit;
(2) updating the orientation of each AHRS sensing unit with EKF;
(3) calculating swing angles based on the data from the two AHRS sensors together with the physical parameters of the crane body.

The main symbols used in the paper and their description have been summarized and listed in Table 1.

### 3.1. Initial Orientation Calculation

A three-axis accelerometer can measure accelerations along each axis in the body frame. In the static state, the sensor is subjected to gravitational acceleration. In this case, we can obtain the angles between the sensing axis and the gravitational force, so that the accelerometers are used to perform leveling. In other words, we can use the three-axis accelerometer to get the pitch and roll angles of the object in the static state. By comparing the outputs of three orthogonal accelerometers with gravity, the tilt angles between the **b** (body) frame and the **l** (level) frame can be determined. The output of the accelerometer in the **b** frame is denoted as $\left(a_{x}^{b},\;a_{y}^{b},\;a_{z}^{b}\right)$, while the gravity in the **l** frame is denoted as (0, 0, *g*). With the orientation transformation matrix from the **b** frame to the **l** frame ${\text{C}}_{b}^{l}$, we have:

(1) $${\left[a_{x}^{b},a_{y}^{b},a_{z}^{b}\right]}^{T}={\left({\text{C}}_{b}^{l}\right)}^{T}{\left[0,0,g\right]}^{T}$$

Since the leveling angles have no relationship to the yaw angle *ψ*, the transformation matrix ${\text{C}}_{b}^{l}$ can be expressed as:

(2) $${\text{C}}_{b}^{l}=\left[\begin{array}{ccc} \cos\theta & \sin\theta \sin\phi & \sin\theta \cos\phi \\ 0 & \cos\phi & -\sin\phi \\ -\sin\theta & \cos\theta \sin\phi & \cos\theta \cos\phi \end{array}\right]$$

Therefore,

(3) $$a_{x}^{b}=-g\sin\theta$$

(4) $$a_{y}^{b}=g\cos\theta \sin\phi$$

(5) $$a_{z}^{b}=g\cos\theta \cos\phi$$

Because the values of $a_{x}^{b}$, $a_{y}^{b}$ and $a_{z}^{b}$ are measured directly by the accelerometer and the gravity is constant (*g* = 9.78 m/s^2^), we can calculate the pitch angle *θ* and roll angle *ϕ* as:

(6) $$\theta =-\arcsin\;\left(\frac{a_{x}^{b}}{g}\right)$$

(7) $$\phi =\arctan\;\left(\frac{a_{y}^{b}}{a_{z}^{b}}\right)$$

Similarly to the accelerometer, for a three-axis magnetometer, as we know that the horizontal component of the earth magnetic field always points north, its direction could be decided by the horizontal components *H_x_* and *H_y_* in the **l** frame, which are the magnetic field along the north and east in the inertial frame. The relationship between the two horizontal components in the **l** frame and the output of magnetometer $\left(h_{x}^{b},\;h_{y}^{b},\;h_{z}^{b}\right)$ in the **b** frame can be expressed as:

(8) $$\left[\begin{array}{c} H_{x}\\ H_{y}\\ 0 \end{array}\right]={\left({\text{C}}_{b}^{l}\right)}^{\text{T}}\left[\begin{array}{c} h_{x}^{b}\\ h_{y}^{b}\\ h_{z}^{b} \end{array}\right]$$

From the formula above, we can get:

(9) $$H_{x}=h_{x}^{b}\cos\theta +h_{y}^{b}\sin\theta \sin\phi +h_{\text{z}}^{b}\sin\theta \cos\phi$$

(10) $$H_{y}=h_{y}^{b}\cos\phi -h_{\text{z}}^{b}\sin\phi$$

Therefore, the yaw angle can be calculated as:

(11) $$\psi =\arctan\;\left(\frac{H_{x}}{H_{y}}\right)$$

In practical engineering applications, the quaternion is used to describe the change of orientation q = [*q*_1_, *q*_2_, *q*_3_, *q*_4_]^T^, since it can avoid singularities. Initial quaternion is obtained in general from the transformation matrix obtained at the initial alignment stage. The conversion from Euler angles to the quaternion is as follows:

(12) $$q_{1}=\cos\frac{\phi }{2}\cos\frac{\theta }{2}\cos\frac{\psi }{2}+\sin\frac{\phi }{2}\sin\frac{\theta }{2}\sin\frac{\psi }{2}$$

(13) $$q_{2}=\sin\frac{\phi }{2}\cos\frac{\theta }{2}\cos\frac{\psi }{2}-\cos\frac{\phi }{2}\sin\frac{\theta }{2}\sin\frac{\psi }{2}$$

(14) $$q_{3}=\cos\frac{\phi }{2}\sin\frac{\theta }{2}\cos\frac{\psi }{2}+\sin\frac{\phi }{2}\cos\frac{\theta }{2}\sin\frac{\psi }{2}$$

(15) $$q_{4}=\cos\frac{\phi }{2}\cos\frac{\theta }{2}\sin\frac{\psi }{2}+\sin\frac{\phi }{2}\sin\frac{\theta }{2}\cos\frac{\psi }{2}$$

### 3.2. Orientation Update

From the characteristics of the three sensors, we can either use a three-axis gyroscope to get the orientation, provided the sensor is perfect enough, or a combination of a three-axis accelerometer and a three-axis magnetometer, if the sensor system is static. However, neither is the gyroscope ideally perfect nor will the sensor system be always static. In this case, one should combine all the three sensors and fuse the outputs from them to estimate the orientation.

Various methods for orientation update have been proposed in robotics and aerospace literature. An excellent review for all of the existing methods in aerospace is given in [24]. The most widely-used method is EKF, and many variants based on EKF are proposed, such as addictive EKF, backwards-smoothing EKF, deterministic EKF-like estimator, interlaced EKF, *etc.* [24]. For a low-cost sensor-based system, the linear single-input-single-output complimentary filters are often used, which provide a tradeoff between accurate short-term gyroscopic integration and good long-term measurements from accelerometers [25]. This method, however, cannot estimate the gyroscope bias compared to EKF [26]. Since the orientation estimation is an observer problem, from the sensor output to infer the true state, nonlinear observer techniques are used in [27] to estimate the bias term. For implementations using embedded processor, there is minimum order Kalman filter, which is comprised of a quaternion measurement step and a linear Kalman filter step to reduce the computation time [28].

In this application, we employed the standard EKF algorithm to estimate and update the orientation based on quaternion representation. Suppose a nonlinear system is modeled by:

$$\begin{array}{c} \dot{\text{x}}=\text{f}\left(\text{x},\text{u},\text{w}\right)\\ \dot{y}=\text{h}\left(x,\text{u},\text{v}\right) \end{array}$$

where **x** ∈ ℝ*^n^* is the state vector that we want to estimate; **u** ∈ ℝ*^p^* is the input; **y** ∈ ℝ*^m^* is measurements from the sensors; **w** and **v** are the process and measurement noise, respectively. Because the nonlinear relation between the noise and the states or measurements is difficult to obtain, we assume that both noises are linear. Moreover, the process is continuous, and the measurement is always discrete with respect to time. Therefore, we can use the hybrid model as follows to examine the orientation estimation problem [29]:

$$\begin{array}{c} \dot{\text{x}}=\text{f}\left(\text{x},\text{u}\right)+\text{w}\left(t\right)\\ {\text{y}}_{k}=\text{h}\left(x_{k},\text{u}\right)+{\text{v}}_{k} \end{array}$$

where we assume vectors **w**(*t*) and **v***_k_* represent white noises with zero mean and covariances **Q** and **R**, respectively.

The EKF linearizes the system at the estimated state, and the process consists of two steps: time update (prediction step) and measurement update (correction step).

In the prediction step, state estimates are projected to a new value by numerically integrating the nonlinear state equations with **w**(*t*) = 0. Similarly, the state error covariance matrix **P** is also propagated. The two predication equations are:

$$\begin{array}{c} \dot{\hat{\text{x}}}=\text{f}\left(\hat{\text{x}},\text{u}\right)\\ \dot{P}=\text{AP}+{\text{PA}}^{T}+\text{Q} \end{array}$$

where:

$$\text{A}={\frac{\partial \text{f}\left(\text{x},\text{u}\right)}{\partial \text{x}}|}_{\text{x}=\hat{\text{x}}}$$

is the linearization of the state equation at current estimated states.

In the correction step, we correct the states with the measurements from the sensors. Denote the propagated states and covariance matrix from the prediction step as **x̂**^−^ and **P**^−^. We first compute the Kalman gain **L** as:

$$\text{L}={\text{P}}^{-}{\text{C}}^{T}{\left({\text{CP}}^{-}{\text{C}}^{T}+\text{R}\right)}^{-1};$$

where:

$$\text{C}={\frac{\partial \text{h}\left(\text{x},\text{u}\right)}{\partial \text{x}}|}_{\text{x}={\hat{\text{x}}}^{-}}$$

is the linearization of the measurement equation at current estimate states. Using the Kalman gain and new measurement **z***_k_*, we can update the states with:

$$\hat{\text{x}}={\hat{\text{x}}}^{-}+\text{L}\left[{\text{z}}_{k}-\text{h}\left({\hat{\text{x}}}^{-},\text{u}\right)\right]$$

Finally, we update the state error covariance matrix by:

$$\text{P}=\left(\text{I}-\text{LC}\right){\text{P}}^{-}$$

where **I** is the identity matrix. From the new states and state error covariance matrix, we can repeat the predication step to form an estimation loop. Based on the EKF presented above, we will try to solve the orientation estimation based on the quaternion.

Using the quaternion approach, we can avoid singularities, which is inevitable in Euler angle methods. In this case, we also assume that the input is the gyro output *ω*, then the process dynamic equation is:

(16) $$\dot{\text{q}}=\frac{1}{2}U\left(\text{q}\right)\omega +\text{w}\left(t\right)$$

where:

$$\begin{array}{cc} U\left(\text{q}\right)=\left(\begin{array}{ccc} {\text{q}}_{4} & -q_{3} & q_{2}\\ q_{3} & q_{4} & -q_{1}\\ -q_{2} & q_{1} & q_{4}\\ -q_{1} & -q_{2} & -q_{3} \end{array}\right), & \left\|\text{q}\right\| \end{array}=1$$

Note that this process dynamics is linear in the states, and we can get the **A** matrix as:

$$\text{A}=\left[\begin{array}{cccc} 0 & \omega_{z} & -\omega_{y} & \omega_{x}\\ -\omega_{z} & 0 & \omega_{x} & \omega_{y}\\ \omega_{y} & -\omega_{x} & 0 & \omega_{z}\\ -\omega_{x} & -\omega_{y} & -\omega_{z} & 0 \end{array}\right]$$

The output equation is [30]:

(17) $$\left[\begin{array}{c} a_{\text{o}}\\ h_{\text{o}} \end{array}\right]=\left[\begin{array}{cc} \text{R}{\left(\text{q}\right)}^{\text{T}} & 0_{3\times 3}\\ 0_{3\times 3} & \text{R}{\left(\text{q}\right)}^{\text{T}} \end{array}\right]\;\left[\begin{array}{c} {\text{a}}_{r}\\ {\text{h}}_{r} \end{array}\right]+\left[\begin{array}{c} {\text{v}}_{a}\\ {\text{v}}_{h} \end{array}\right]$$

where **a_o_** and **h_o_**, **a***_r_* and **h***_r_*, **v***_a_* and **v***_h_* are the outputs, reference outputs and measurement noise for the accelerometer and magnetometer, respectively. **R**(**q**) is the rotational matrix from body frame to inertial frame obtained from the quaternion:

$$\text{R}\left(\text{q}\right)=\left[\begin{array}{ccc} q_{4}^{2}+q_{1}^{2}-q_{2}^{2}-q_{3}^{2} & 2\left(q_{1}q_{2}-q_{3}q_{4}\right) & 2\left(q_{1}q_{3}+q_{2}q_{4}\right)\\ 2\left(q_{1}q_{2}+q_{3}q_{4}\right) & q_{4}^{2}-q_{1}^{2}+q_{2}^{2}-q_{3}^{2} & 2\left(q_{2}q_{3}-q_{1}q_{4}\right)\\ 2\left(q_{1}q_{3}-q_{2}q_{4}\right) & 2\left(q_{2}q_{3}+q_{1}q_{4}\right) & q_{4}^{2}+q_{1}^{2}-q_{2}^{2}+q_{3}^{2} \end{array}\right]$$

From the structure of the output dynamics, we can implement the correction step in two sub-steps, one for the accelerometer and another one for the magnetometer.

To view the updating quaternion in terms of Euler angles, one can transform the quaternion to Euler angles, which can be accomplished by:

(18) $$\left[\begin{array}{c} \phi \\ \theta \\ \psi \end{array}\right]=\left[\begin{array}{c} \arctan2\left(2\left(q_{4}q_{1}+q_{2}q_{3}\right),1-2\left(q_{1}^{2}+q_{2}^{2}\right)\right)\\ \arcsin\left(2\left(q_{4}q_{2}-q_{3}q_{1}\right)\right)\\ \arctan2\left(2\left(q_{4}q_{3}+q_{1}q_{2}\right),1-2\left(q_{2}^{2}+q_{3}^{2}\right)\right) \end{array}\right]$$

For the transformation, the angles for *ϕ* and *ψ* may be subjected to an abrupt change near −*π* or *π*. This is because the arctan 2 function generates an angle between [−*π*, *π*].

**Figure 3.**
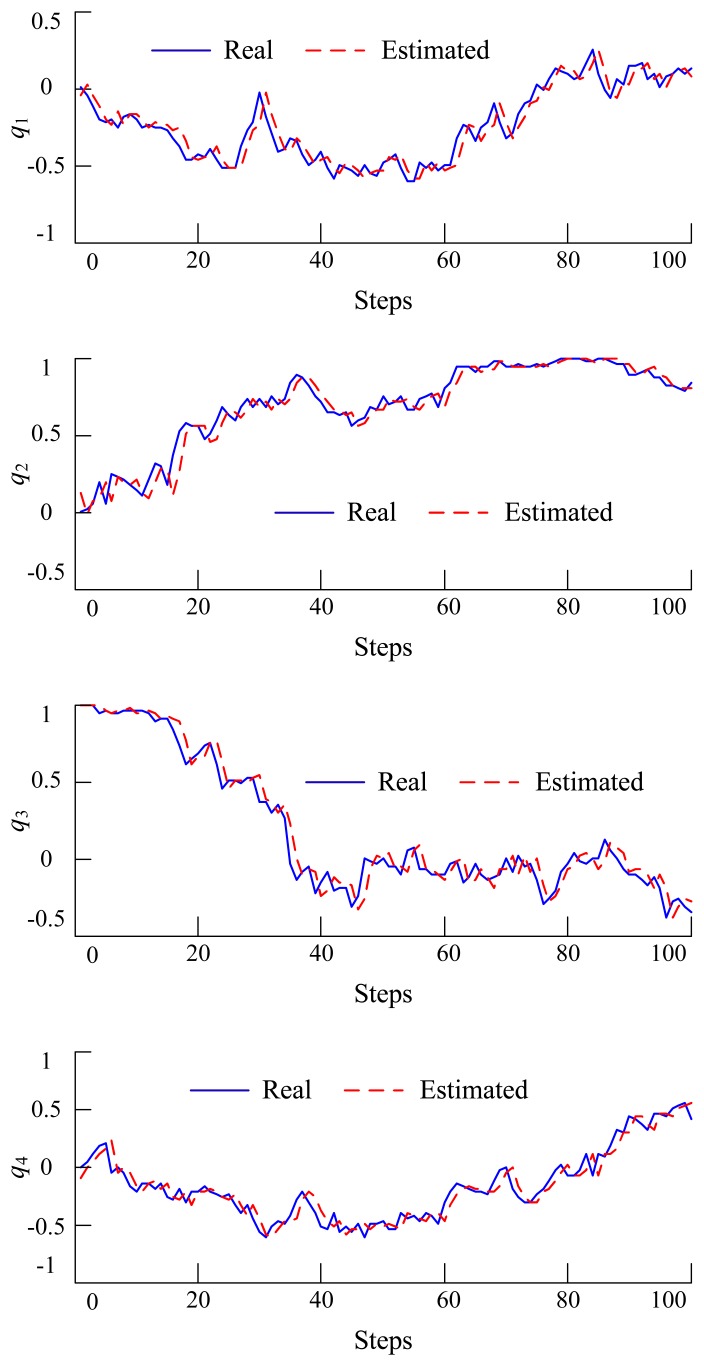
Simulation results of EKF estimation based on the quaternion.

To verify whether the above filtering algorithm will work or not, a simulation in MATLAB has been performed. Additionally, the simulation results are shown in Figure 3, where the solid blue line is the true value, while the dashed red line is the estimated value. From the figure, we can see that the estimated value tracks the true state value very well.

### 3.3. Swing Angles Calculation

**Figure 4.**
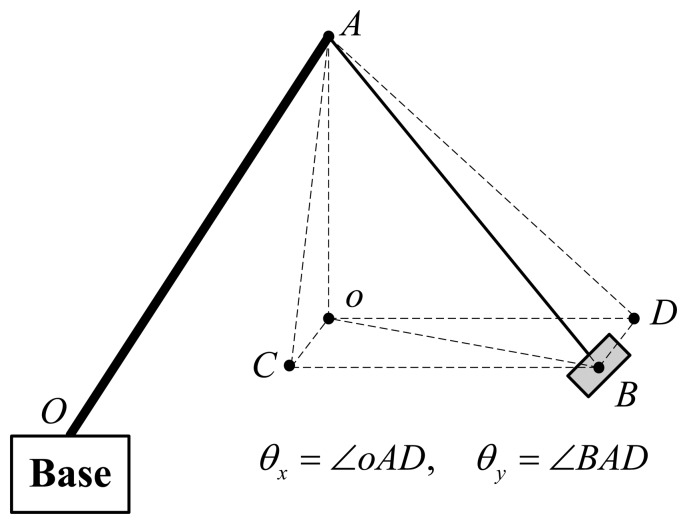
Angles' description.

To calculate the swing angles *θ_x_* and *θ_y_*, the special case that the payload has no rotation about the rope is considered at first. The geometry description of the swing angles and AHRS angles can be depicted with Figure 4. In Figure 4, plane *oCDB* is horizontal; point *o* is the projection of point *A* (top tip of the jib) on plane *oCDB*; point *B* is the center of the payload, as well as the embedded Sensing Unit 2; *BC*⊥*oCA*, *BD*⊥*oDA*. Therefore, the spatial swing angles can be described as *θ_x_* = ∠*oAD* and *θ_y_* = ∠*BAD*. There are two assumptions:

(1) The rope line of the crane is always vertical to the plane of the payload, as well as the embedded Sensing Unit 2;
(2) The payload, as well as the embedded Sensing Unit 2 does not rotate about the rope of the crane.

Based on the above two assumptions, we have ∠*oAB* = *θ*, where *θ* is the pitch angle measured by Sensing Unit 2 and ∠*BoD* = Δ*ψ* where Δ*ψ* is the difference of the yaw angle calculated according to the yaw angles measured by Sensing Unit 1 and Sensing Unit 2.

According to the geometric relationship, as shown in Figure 4, we have:

(19) $$\theta_{x}=\pi /2-∠Α\text{Do}$$

(20) sin∠oBA=sin∠ABD⋅sin∠ADo

(21) $$\theta_{y}=\pi /2-∠\text{ABD}$$

(22) cos∠ABD=cos∠oBA⋅cos∠oBD

where ∠*oBD* = *π*/2 − Δ*ψ* and ∠*oBA* = *π*/2 − *θ*. Therefore, the angle *θ_x_* and *θ_y_* can be calculated as:

(23) $$\theta_{y}=\arcsin\;\left(\sin\theta \cdot \sin\Delta \psi \right)$$

(24) $$\theta_{x}=\arccos\;\left(\cos\theta \cdot \sec\theta_{y}\right)$$

For general cases with pitch, roll and yaw angle (*θ, ϕ, ψ*) measured by the MEMS-based AHRS sensor embedded in the payload, the special case can be achieved by rotating the AHRS sensor with respect to axis *z* in the body frame with an angle *α*. Furthermore, corresponding pitch and yaw angles in the special case are denoted by *θ′* and *ψ′*, respectively The relationship between the general case and special case can be represented with the orientation matrix **R**(*θ, ϕ, ψ*) as:

(25) $$\left[\begin{array}{ccc} C_{\alpha } & S_{\alpha } & 0\\ -S_{\alpha } & C_{\alpha } & 0\\ 0 & 0 & 1 \end{array}\right]\cdot \text{R}\left(\theta ,\phi ,\psi \right)=\left[\begin{array}{ccc} C_{\theta \prime }C_{\psi \prime } & C_{\theta \prime }S_{\psi \prime } & -S_{\theta \prime }\\ -S_{\psi \prime } & C_{\psi \prime } & 0\\ S_{\theta \prime }C_{\psi \prime } & S_{\theta \prime }S_{\psi \prime } & C_{\theta \prime } \end{array}\right]$$

(26) $$\text{R}\left(\theta ,\phi ,\psi \right)=\left[\begin{array}{ccc} C_{\theta }C_{\psi } & C_{\theta }S_{\psi } & -S_{\theta }\\ S_{\phi }S_{\theta }C_{\psi }-C_{\phi }S_{\psi } & S_{\phi }S_{\theta }S_{\psi }+C_{\phi }C_{\psi } & S_{\phi }C_{\theta }\\ C_{\phi }S_{\theta }C_{\psi }+S_{\phi }S_{\psi } & C_{\phi }S_{\theta }S_{\psi }-S_{\theta }C_{\psi } & C_{\phi }C_{\theta } \end{array}\right]$$

where *C_α_* = cos *α*, *S_α_* = sin *α* and a similar denotation for the others. Therefore, the corresponding pitch angle *θ′* and yaw angle *ψ′* in the special case can be calculated as:

(27) θ′=arccos(cosϕ⋅cosθ)

(28) $$\psi \prime =\arccos\;\left(\frac{S_{\phi }S_{\psi }S_{\theta \prime }}{{S_{\theta }}^{2}+{S_{\phi }}^{2}{C_{\theta }}^{2}}+\frac{S_{\theta }S_{\theta \prime }C_{\phi }C_{\psi }}{{S_{\theta }}^{2}+{S_{\phi }}^{2}{C_{\theta }}^{2}}\right)$$

Now, we can calculate the angles *θ_y_* and *θ_x_* with Equations (23) and (24) based on the new *θ′* and Δ*ψ′* = *ψ′* − *ψ*_0_, where *ψ*_0_ is the yaw angle measured by Sensing Unit 1.

## 4. Experiments

**Figure 5.**
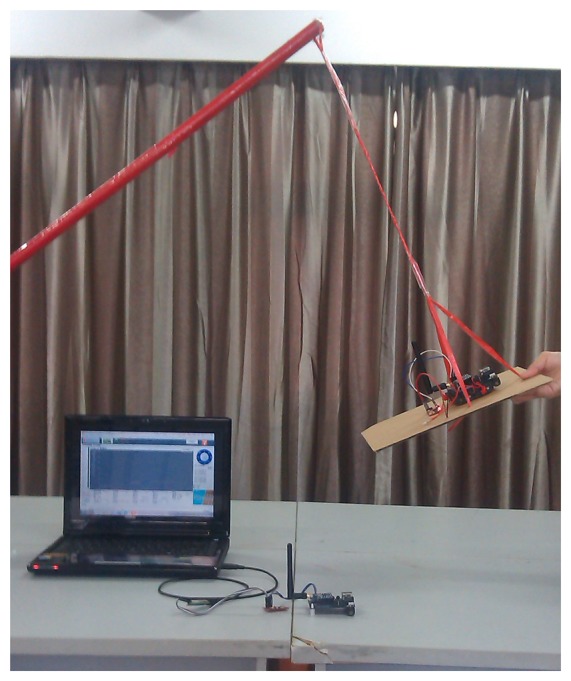
Photo of an experiment.

**Figure 6.**
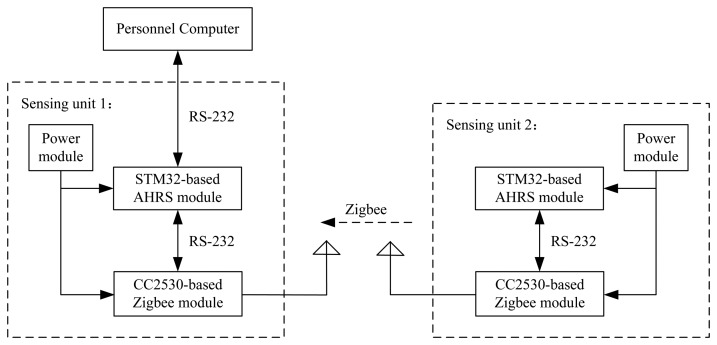
Diagram of the experimental setup.

Experiments have been performed to verify the designed swing angle measurement system. A picture of an experiment is shown in Figure 5, and the electrical diagram of the experiment is illustrated in Figure 6. Each sensing unit consists of three main modules, *i.e.*, power module, STM32-based AHRS module and CC2530-based ZigBee module. The STM32-based AHRS module of Sensing Unit 2, as shown in Figure 1, calculated the pitch, roll and yaw angles of the payload in real time based on the algorithms developed in Section 3; and the calculated orientation of the payload is sent to the CC2530-based ZigBee module with serial communication RS-232. The wireless communication module will send the orientation of the payload to Sensing Unit 1, as shown in Figure 1, with the ZigBee communication module. Similarly, the orientation of the crane base is achieved with the STM32-based AHRS module of Sensing Unit 1. Therefore, the swing angles of the payload will be calculated in the STM32-based unit, and then, they will be sent to the control box of the crane with serial communication. As in the experiment, a laptop is used to acquire the angle data and to mimic the control box of the crane system.

**Figure 7.**
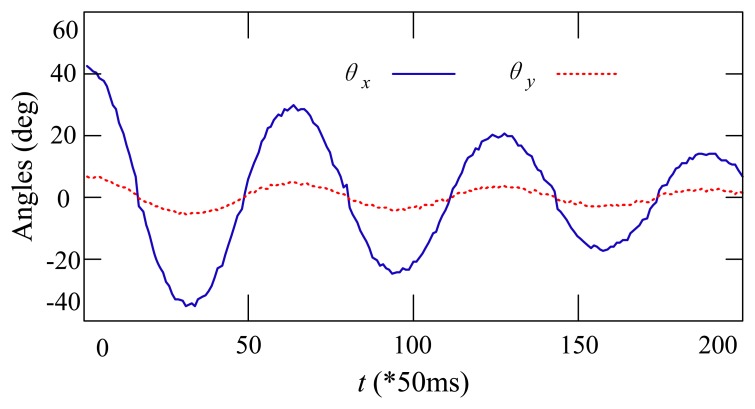
Experimental results of the dynamic angle measurement.

Static and dynamic swing angle measurements based on the developed system were performed. Table 2 shows the static experiment results of the swing angles measurement. During the experiments, the payload was fixed to four typical angles by hand. Comparing the measured results on four typical swing angles of the payload, the measured error increases as the measured angle increases. When the payload has small swing angles, the error of the measured angles is less than one degree. However, when the measured angles become as large as 60 degrees, the measured error is larger than two degrees. The performance of the dynamic angle measurement is shown in Figure 7. During the experiment, the payload was initialized by hand to a random position and then left to swing freely. One can see that the curves of the measured swing angles are smooth and that the swing motion is being damped because of friction.

**Figure 8.**
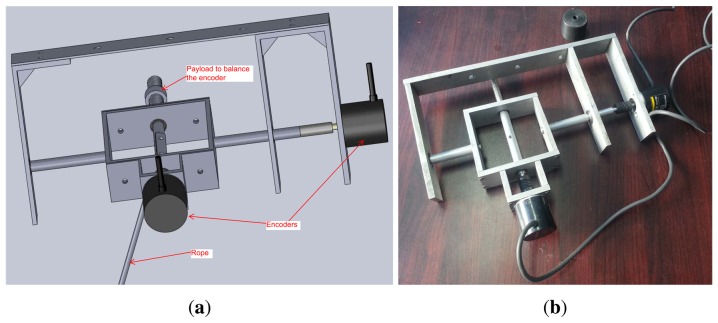
Swing angle measuring mechanism. (**a**) CAD design of swing angle measuring mechanism based on encoders; (**b**) Picture of the swing angle measuring mechanism based on encoders.

**Figure 9.**
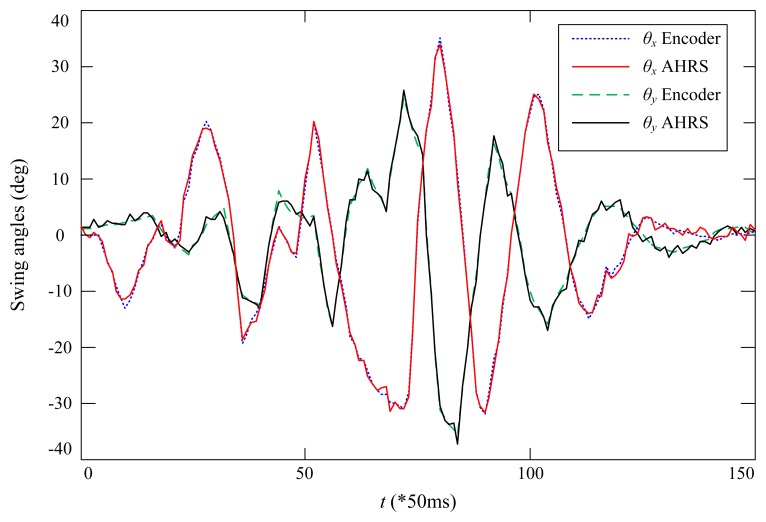
Measured swing angles based on the proposed wireless AHRS-based scheme and optical encoders.

**Figure 10.**
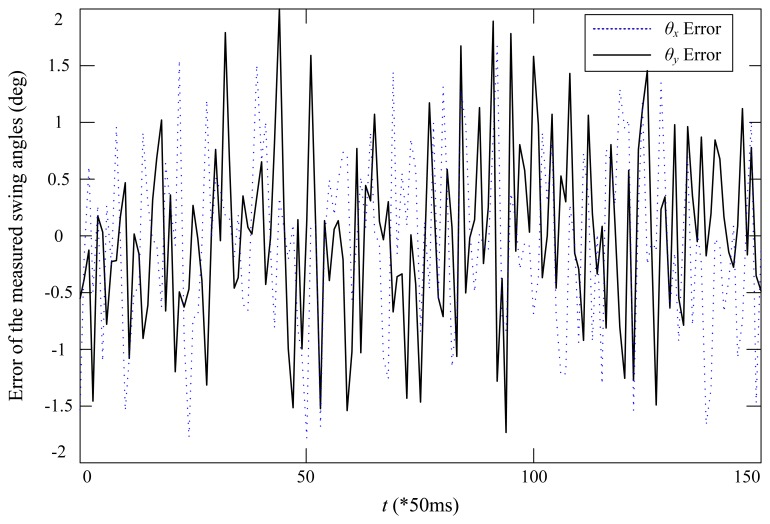
Error of the measured swing angles based on the proposed wireless AHRS-based scheme compared to the optical encoders.

To further examine the accuracy of the developed swing angle measurement system, two high resolution encoders, E6B2-CWZ6C, with 2000 pulses per round from Omron have been used to detect the two angles. The CAD design and the prototype of the encoder-based swing angle measuring mechanism are shown in Figure 8. The payload was moved by hand for several seconds, and the recorded measured angles are shown in Figure 9. One can see that the swing angles measured from the developed AHRS-based wireless scheme using MEMS devices are very close to those measured from the encoders. The displacement errors of the two angles are shown in Figure 10. The mean errors of swing angles *θ_x_* and *θ_y_* are both 0.61 degrees; and the maximum errors of swing angles *θ_x_* and *θ_y_* are 1.79 degrees and 2.0 degrees, respectively. Although the maximum measuring error is as big as 2.0 degrees by using the developed scheme compared to the encoders scheme, the mean error is steadily around 0.6 degrees, which could be accurate enough for monitoring and anti-swing control in crane systems. Moreover, it is impractical to use the encoder-based scheme in a very large crane system, because additional complicated mechanisms have to be designed and added to the jib of the crane system. However, the developed scheme is low cost and easy to embed in real crane systems.

It should be noted that the mean error of 0.61 degrees was achieved under the lab experimental conditions: around 25 Centigrade, only lights, several computers and one air-conditioning are running. In real industrial environments, the system based on the three MEMS devices should be calibrated in order to obtain the same level of accuracy, because the signals based on these MEMS devices will be disturbed, especially the magnetic signals [30]. Additionally, while transforming the experimental sensing unit to the real environmental implementation, the AHRS module and ZigBee module, as shown in Figure 6, could be redesigned and integrated as one module.

## 5. Conclusions and Future Work

Measuring the swing angles of different crane systems effectively is helpful to improve the safety and efficiency of the crane systems. The paper proposed a wireless AHRS-based swing angle measurement system by using MEMS devices and ZigBee wireless communication. Preliminary experiments have demonstrated the feasibility of the proposed scheme and confirmed that the mean error of the measured swing angles is no more than 0.7 degrees. Since all of the devices have small dimensions and are low cost, the developed swing angle measurement system is low cost and easy to embed in real crane systems. The wireless feature of the measurement system means that it can be installed conveniently and will not be influenced by the weather environment. Therefore, the developed swing angle measurement system could be widely applied for crane systems.

The experimental results in this paper were achieved in the laboratory. Additionally, the signals of the MEMS devices would be disturbed in real industrial environments. Future work will focus on verifying the scheme in real applications and improving the accuracy of the measurement system.

## Figures and Tables

**Table 1.** Summary of notation.

**Symbol**	**Description**
*θ_x_*	planar swing angle describing the far and near swing of a payload
*θ_y_*	planar swing angle describing the left and right swing of a payload
*θ*	general pitch angle
*ϕ*	general roll angle
*ψ*	general yaw angle
*θ****′***	corresponding pitch angle in the special case
*ψ′*	corresponding yaw angle in the special case
Δ*ψ*	difference of yaw angles measured by the two sensing units
Δ*ψ′*	difference of yaw angles measured by the two sensing units in the special case
**q**	quaternion used to describe the orientation q = [*q*_1_,*q*_2_,*q*_3_, *q*_4_]^T^
**a**	three-axis accelerometer output having three items *a_x_, a_y_, a_z_*
**h**	three-axis magnetometer output having three items *h_x_,h_y_, h_z_*
*ω*	three-axis gyroscope output having three items ω*_x_*,ω*_y_, ω_z_*
$a_{x}^{b},a_{y}^{b},a_{z}^{b}$	output of the three-axis accelerometer in the **b** (body) frame
${\text{C}}_{b}^{l}$	transformation matrix from the **b** (body) frame to the **l** (level) frame
*g*	gravity constant
$h_{x}^{b},h_{y}^{b},h_{z}^{b}$	output of the three-axis magnetometer in the **b** (body) frame
*H_x_, Hy*	magnetic field along the north and east in the inertial frame
**x**	state vector of a system
**x**	estimated state vector of a system
**x̂**^−^	propagated states from the prediction step
**y**	output vector of a system
**u**	input vector of a system
**w**	process noise vector of a system
**v**	measurement noise vector of a system
**Q**	covariance matrix of the process noise
**R**	covariance matrix of the measurement noise
**P**	covariance matrix of the state error
**P**^−^	propagated covariance matrix from the prediction step
**A**	linearization of the state matrix at current estimated states
**L**	Kalman gain
**C**	linearization of the measurement matrix at current estimate states
**I**	identity matrix
**a_o_**	output of the accelerometer during orientation updating
**h_o_**	output of the magnetometer during orientation updating
**a_r_**	reference output of the accelerometer during orientation update
**h_r_**	reference output of the magnetometer during orientation update
**v_a_**	measurement noise of the accelerometer during orientation update
**v_h_**	measurement noise of the magnetometer during orientation update
**R**(**q**)	rotational matrix from body frame to inertial frame in quaternion
**R**(*θ, ϕ, ψ*)	rotational matrix from body frame to inertial frame in Euler angles

**Table 2.** Static angles measurement.

**Angles (deg)**	**Real Angle**	**0**	**30**	**45**	**60**
*θ_x_*	Measured Angle	0.4	28.8	46.2	62.6
*θ_y_*	Measured Angle	0.3	29.5	44.3	61.7
